# Nanocarriers surface engineered with cell membranes for cancer targeted chemotherapy

**DOI:** 10.1186/s12951-022-01251-w

**Published:** 2022-01-21

**Authors:** Wen Lei, Chen Yang, Yi Wu, Guoqing Ru, Xianglei He, Xiangmin Tong, Shibing Wang

**Affiliations:** 1 Department of Hematology, The Second Affiliated Hospital, College of Medicine, Zhejiang University, Hangzhou, Zhejiang 310009 People’s Republic of China; 2 Department of Ultrasonography, Zhejiang Provincial People’s Hospital, People’s Hospital of Hangzhou Medical College, Hangzhou, Zhejiang 310014 People’s Republic of China; 3 Phase I Clinical Research Center, Zhejiang Provincial People’s Hospital, People’s Hospital of Hangzhou Medical College, Hangzhou, Zhejiang 310014 People’s Republic of China; 4 Departments of Pathology, Zhejiang Provincial People’s Hospital, People’s Hospital of Hangzhou Medical College, Hangzhou, Zhejiang 310014 People’s Republic of China; 5 Cancer Center, Key Laboratory of Tumor Molecular Diagnosis and Individualized Medicine of Zhejiang Province, Zhejiang Provincial People’s Hospital, Affiliated People’s Hospital, Hangzhou Medical College, Hangzhou, Zhejiang 310014 People’s Republic of China

**Keywords:** Nanocarriers, Cell membrane, Cancer, Chemotherapy, Targeted drug delivery

## Abstract

**Background:**

Inspired by nature, the biomimetic approach has been incorporated into drug nanocarriers for cancer targeted chemotherapy. The nanocarriers are cloaked in cell membranes, which enables them to incorporate the functions of natural cells.

**Key scientific concepts of review:**

Nanocarriers surface engineered with cell membranes have emerged as a fascinating source of materials for cancer targeted chemotherapy. A distinctive characteristic of cell membrane-coated nanocarriers (CMCNs) is that they include carbohydrates, proteins, and lipids, in addition to being biocompatible. CMCNs are capable of interacting with the complicated biological milieu of the tumor because they contain the signaling networks and intrinsic functions of their parent cells. Numerous cell membranes have been investigated for the purpose of masking nanocarriers with membranes, and various tumor-targeting methods have been devised to improve cancer targeted chemotherapy. Moreover, the diverse structure of the membrane from different cell sources broadens the spectrum of CMCNs and offers an entirely new class of drug-delivery systems.

**Aim of review:**

This review will describe the manufacturing processes for CMCNs and the therapeutic uses for different kinds of cell membrane-coated nanocarrier-based drug delivery systems, as well as addressing obstacles and future prospects.

**Graphical Abstract:**

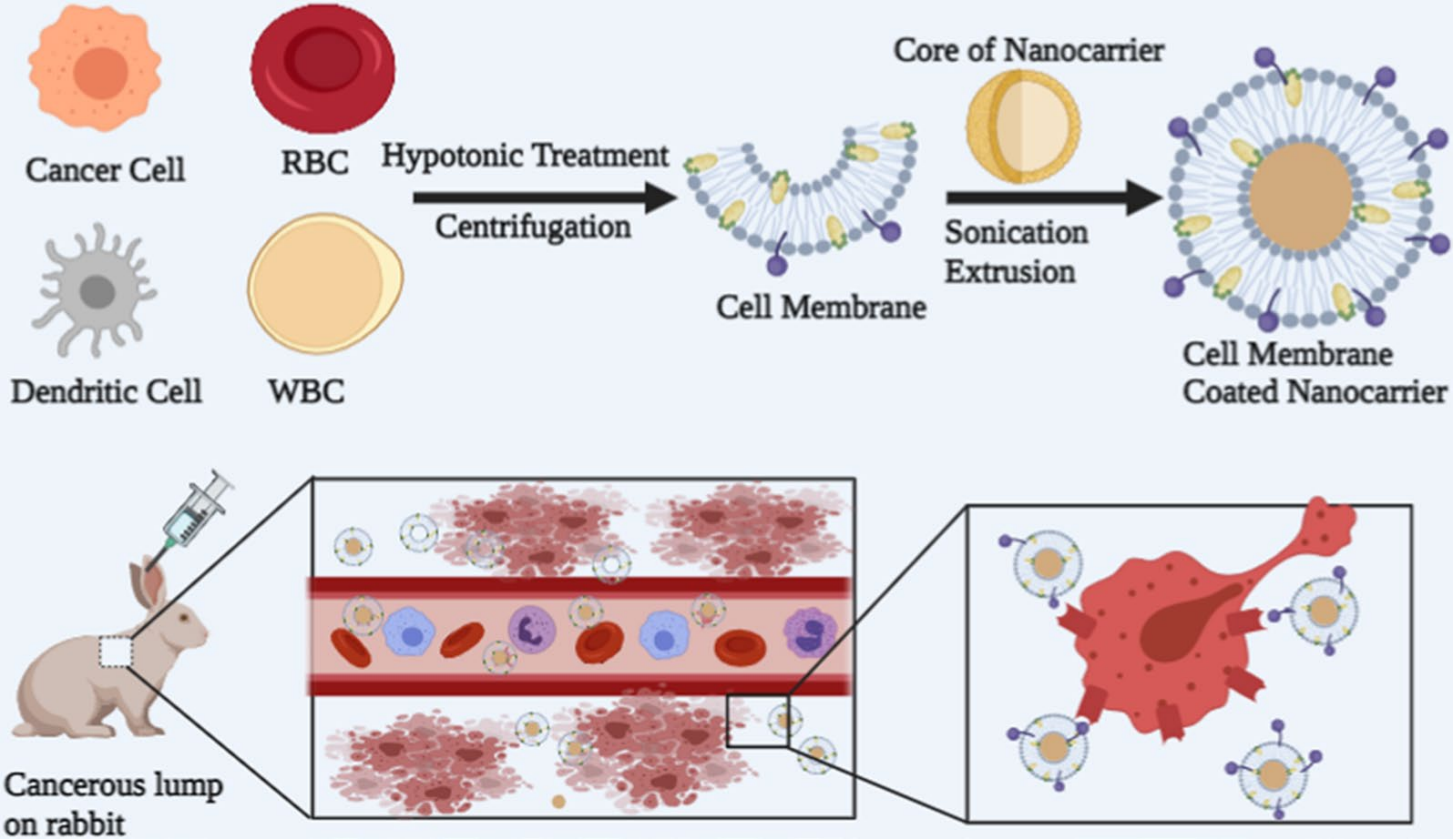

## Background

Cancer has been a worldwide concern for a long period of time and is the second largest cause of mortality [1]. Conventional chemotherapy, as one of the most frequently used methods for cancer treatment, remains unsatisfactory owing to the significant side effects and the poor targeting ability of anti-cancer drugs [2]. To overcome these issues, significant research and development has been conducted on targeted drug delivery systems (TDDS), particularly nanocarrier-based TDDS [3]. The benefits of nanocarriers, which include the ability to be modified, a large capacity for drug loading, and tunable physiochemical characteristics, make them ideal for encapsulating anti-cancer drugs and altering their stability, solubility, and in vivo behaviour [4]. Nevertheless, surface modification of nanocarriers may enhance their blood circulation and enable more precise targeting, thus increasing effectiveness while trying to minimize side effects [5]. However, there are also many disadvantages that make it difficult for nanocarriers to live up to clinical standards. The immune system recognizes and eliminates the majority of nanocarriers as foreign substances. Since the polyethylene glycol (PEG), a hydrophilic polymer, was initially incorporated into a protein medication [6], PEGylation has been the most frequently utilized modification technique in drug delivery applications [7]. Additionally, the targeted capacity of nanocarriers was highly reliant on the surface modification, which was challenging to manufacture and accomplish [8]. As a result, TDDS delivered through nanoparticles has not yet achieved its full therapeutic potential.

**Fig. 1:**
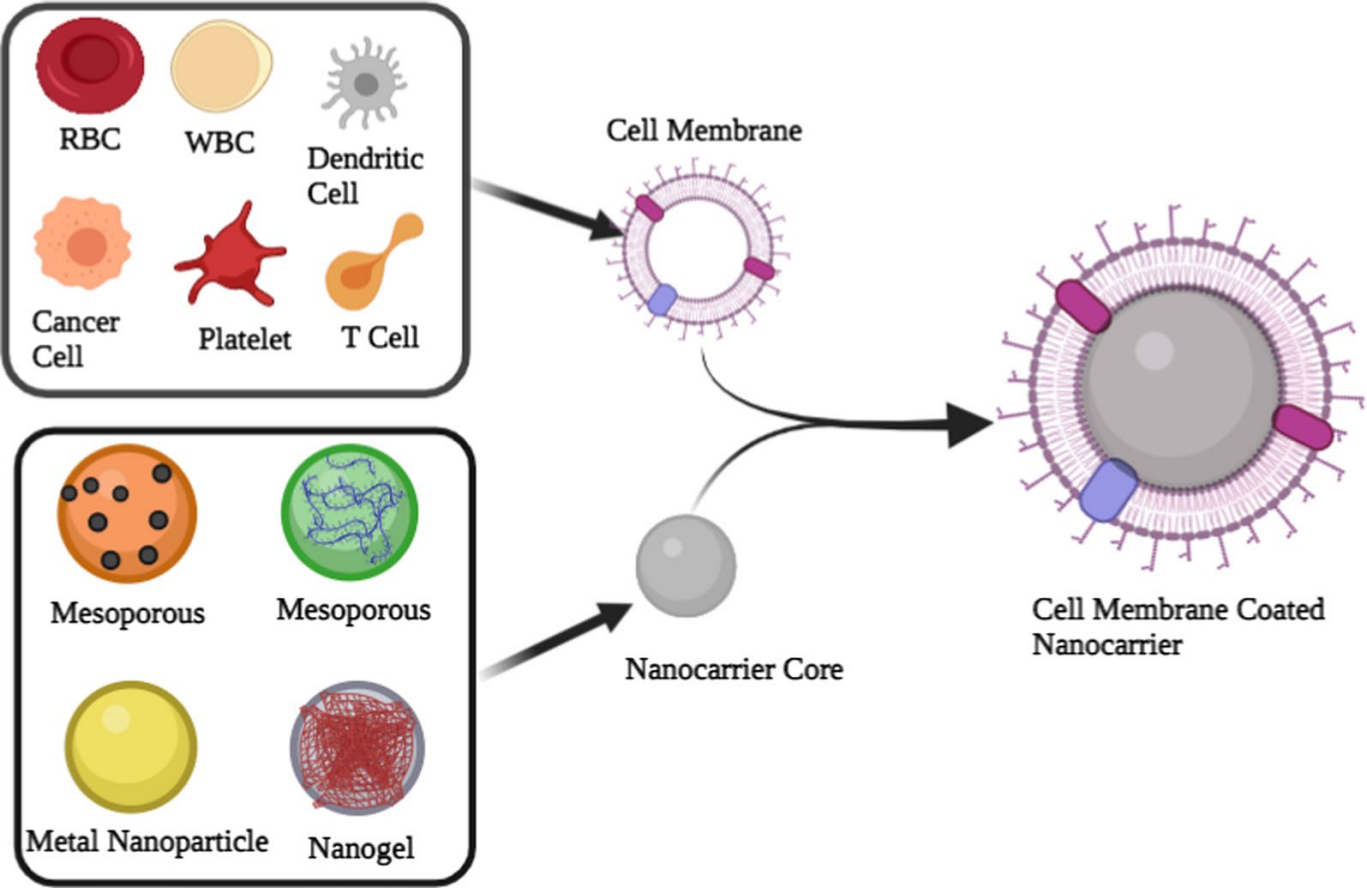
Nanocarriers with a cell membrane coating for cancer drug delivery. Different types of cell membranes are used to encapsulate various types of nanocarrier core for cancer treatment

The drug-delivery system's (DDSs) technology continues to advance, making it possible to administer more potent drugs [9]. Drug research efforts are significantly aided by therapeutic compounds’ capacity to remain intact in a hostile extracellular milieu [10]. In this connection, efforts to reduce immunogenicity and improve biopharmaceutical stability through modification of biopharmaceuticals have increased [11]. Cells in the early 1980s were used as drug delivery vehicles, which substantially increased the drugs' retention and targeting capabilities [12]. Despite the increasing use of live cell-based carriers, several shortcomings persist. One major concern is passenger drug activity, as drugs may be digested by the cell carrier’s lysosomes [13]. Moreover, drug release is difficult to control due to exocytosis or leakage during transport [14]. Faced with these challenges, scientists recently discovered a natural way to design biomimetic cell membrane nanocarriers. At first, the biomimetic cell membrane nanocarriers were made from a poly (lactic-*co*-glycolic acid) (PLGA) core and a red blood cell (RBC) membrane shell, using a co-extrusion process [15]. Then, different cell membrane-coated nanocarriers (CMCNs) were explored with different nanocarrier cores and membrane materials. The incorporation of nanocarriers into the cell membrane merges the advantages of material science and biomimicry. It is important to note that CMCNs can be portrayed as autogenous cells to prolong blood circulation time and avoid immune system elimination, both of which are required for the enhanced permeability and retention (EPR) effect of cancer targeted chemotherapy [16] (Fig. 1).

Moreover, different cell membranes may confer different functions on CMCNs, resulting in varying in vivo behaviour. Biomimetic technology, a relatively new procedure, satisfies these requirements and is currently being used in designing drug nanocarriers [17, 18]. By drawing inspiration from nature that comprises biological elements and living matter, this technology aims to overcome the shortcomings of current drug delivery systems. An ideal biomimetic delivery system exploits pathogens’ immune evasion and intracellular uptake tactics. However, delivery systems derived from pathogens continue to raise safety concerns, including immunogenicity and virulence [19].

### Advantages of CMCNs based drug delivery systems

CMCNs have notably contributed to suppressing drug resistance in the use of nanocarriers for cancer therapies. Biomimetic CMCNs possess special characteristics, such as prolonged drug delivery, immunological evasion, homotypic targeting, longer blood circulation, and specific ligand/receptor recognition. To get beyond the restrictions of cell toxicity, differentiation, and sensitivity in cell-based delivery systems, CMCNs utilize therapeutically relevant cell membrane proteins as an alternative to the whole cell. Because a longer circulation time benefits with the potential of sustained drug delivery and increases the probability of sustained distribution into the circulation [20]. The biomimetic CMCNs provide bio-modulation and more control in this regard. The CMCNs prepared by coating RBC membrane on PLGA nanocarriers improved the nanocarriers’ retention in the blood by 72 hours, compared to 15.8 hours for typical synthetic stealth nanocarriers [15]. Moreover, PLGA nanocarriers with a fluorocarbon core masked in an RBC membrane were used for delivering oxygen to solid tumours, demonstrating another application of CMCNs delivery to improve blood circulation time via the EPR approach [21].

The reduced immunogenic characteristics of cancer cell membranes and their homing abilities improve targeted drug delivery at the cancer site. In this respect, Cao et al. investigated the interaction between VCAM-1 of metastatic cancer cells with macrophage α4 proteins to transport cytotoxic anticancer drugs to the lungs [22]. Using the adhesion characteristics of galectin-3 and T antigen in cancer cell membranes, Fang et al. demonstrated homotypic tumour targeting [23]. Furthermore, when compared to other active targeting methods, incorporating iron oxide nanocarriers into fractured cancer cell membranes for tumor targeting demonstrated superior homing to homologous tumors in vivo [24]. Recently designed leuko-like vectors (LLV) targeting metastatic niches utilizing neutrophil membrane-coated nanocarriers have shown a two- to threefold increase in metastatic foci accumulation compared to PLGA-PEG nanocarriers and bare nanocarriers, respectively [25]. This affinity for metastatic niches is enhanced by the presence of N cadherin, Mac-1, and other sticky proteins produced on neutrophil membranes on CMCNs, as opposed to the usual PEG coating employed to prolong circulation half-life and prevent clearance [26]. Interestingly, PLGA nanocarriers coated with T lymphocyte membranes were also capable of retaining their lymphocyte coating and evading lysosome sequestration, while bare nanocarriers were caught in endolysosomal compartments prone to breakdown in in vivo [27]. Moreover, this study also discovered that T lymphocyte-coated nanocarriers had a twofold increase in particle density throughout tumours in mice when compared to naked nanocarriers. Numerous additional research groups are attempting to harness the cell membrane’s inherent properties to create biomimetic drug carriers for cancer treatments.

## Considerations of CMCNs

### Choice of cell membrane

A thorough understanding of the homeostasis, function, and structure of cells in their complex physiological context provides key hints for better biointerfacing of synthetic DDSs [28]. A delivery system with the ability to protect cargo and carry cell features like autonomous activity, compartmentalization, flexibility, and form can be more convenient and beneficial than other delivery systems. The cell membrane repeats the surface functionality of cells and extracellular vesicles as it is the fundamental structural component of them. It is primarily made up of carbohydrates, proteins, and lipids, and it interacts with the environment to survive and grow [29]. Carbohydrates play a part in cellular recognition, whereas proteins are responsible for adhesion and signaling, and lipid bilayer formation combines structural fluidity and stiffness [30–32]. Cell membranes can be differentiated based on the properties and composition of these three components in them. The potential to profit from native cell membrane functions has sparked tremendous scientific interest in coating nanocarriers. If done appropriately, the cell membrane retains its capability, and its coating enhances biointerfacing.

The selection of the appropriate cell type or cell membrane is crucial for ensuring site-specific distribution and targeting as well as for minimizing adverse interactions with complementing systems in vivo. Every cell type has unique biological features, making them suitable for certain therapeutic applications such as infectious diseases, inflammatory diseases, cancer, and personalized therapy [33]. For example, the membrane of RBCs is rich with glycophorins that play a key role in attracting pathogens to their surface and killing them via oxytosis [32]. The application of an RBC membrane to the nanocarriers thereby increases pathogen clearance, long-term circulation, and cell viability. Platelets interact with injured endothelial cells and engage with immune cells to mobilize them toward the inflamed site [34]. As a result, covering the nanocarriers with the platelet membranes allows for selective adherence to tumour tissues or wounded vessels, targeting circulatory tumour cells, pathogen eradication, and the capability to elude detection by macrophages. Similarly, macrophage membranes like other leukocytes carry adhesion molecules like VLA-4, LFA-1, PSGL-1, L-selectin, and P-selectin that help with cell adherence [35]. Thus, coating the nanocarrier with macrophage membrane has the ability to bind pathogens while avoiding macrophage recognition and offering active targeting at the cancer site. Moreover, tumor-specific adhesion molecules and antigens such as mucoprotein-1, epithelial-adhesion molecules, lymphocyte-homing receptors (like CD44), galectin-3, integrins, and cadherins are overexpressed on the surface of cancer cell membranes [36]. These antigens and adhesion molecules play a critical role in the contacts among cells and between cells and the surrounding tissue matrix. Generally, cancer cell membranes can cling to their homologous cells [37]. So, wrapping a nanocarrier with a cancer cell membrane prevents macrophage detection, allowing for homotypic tumour targeting, and contributes to the design of personalized cancer therapy.

### Cell source

In order to use maximum cell membrane properties, it is essential to consider the state, form, and source of the cell. In this connection, Evangelopoulos et al. demonstrated that the cell source determines the immunogenicity of biomimetic nanocarriers [38]. They studied multilayer cell membrane generated vesicles from various sources for phagocytosis, opsonization, and targeting of inflamed regions. Literature showed that the use of a syngeneic cell membrane coating increased the avoidance of absorption by the liver and immunological repertoire cells [38]. To isolate the cell membrane for the coating of nanocarriers, it is preferred to choose homotypic cells in a healthy state and nourishing phase. The real therapeutic effectiveness of CMCNs requires homogeneity of the cell population. To fulfil this requirement, quantification or expression levels of specific surface markers (e.g., receptors or ligands) plays a dominant role. For this purpose, flow cytometry, Blot Western, and SDS-PAGE techniques can be used to evaluate the cellular state and homogeneity of cell membranes [39]. Identification of cell biomarkers and other ligands for signal transduction, targeting, or any other approach would enhance translational effects.

### Membrane stability

CMCNs are preferred for use over targeting nanocarriers prepared via a bottom-up approach because they possess numerous characteristics, including signal transduction, immune evasion, targeting, and therapeutic advantages. To maximize the therapeutic potential of CMCNs, the structural and functional characteristics of the cell membrane should be preserved prior to coating drug carriers. The cell membrane’s stability is critical in determining the overall durability of CMCNs. The microenvironment of tissue and circulation naturally creates torque and shear forces on cells and nanocarriers. Cells survive with these forces and respond to them by actively modulating their cytoskeleton-membrane interactions, lipid profile, ligand density, and ligand concentration. For example, the interaction of intracellular proteins with the cell membrane strengthens the reliability of natural cells. During the isolation of the membrane, some key stability regulators of the cell membrane may be lost or changed. As a result, determining the overall membrane stability of CMCNs becomes critical before moving further with biomimetic-based treatment [40]. Numerous techniques for determining the stability of membrane structures are described in the literature. For visualizing the structural integrity and morphology of cell membranes, advanced fluorescence, lipophilic dye enhanced, Cryo-TEM, and spectrophotometric techniques, for example, are all extremely useful [41]. When it comes to the mechanical or elastic integrity of membranes, ektacytometry may be the best tool for determining membrane elongation in dynamic shear stress [42]. Additionally, the source of lipid composition in the cell also influences the overall stability of CMCNs. In one study comparing the lipidomic profiles of cells, a higher proportion of unsaturated phospholipids was observed in primary cell cultures than other cultured. X-ray scattering, FTIR, and colorimetric lipid assays are all useful tools for assessing the qualitative composition of phospholipids [43].

### Membrane-related proteins

The CMCNs interact with the local environment of tissues and cells through proteins present on the cell membranes. So, the appropriate membrane proteins must be kept up in the cell culture. Several transfection and chemical signaling methods may be used to regulate protein expression and cellular states in culture. In fact, long-term cell growth of some cell types may alter their desirable characteristics for CMCNs applications. For example, the culture condition affects the phenotypes of mesenchymal stem cells, which vary across individuals, cell groups, and even batches. The expansion of mesenchymal stem cells in in vitro not only alters mRNA expression patterns but also affects the surface proteins involved in migration and adhesion (e.g., C-met/HGF, CXCR7, CXCR4, etc.) [44]. In the case of nanocarriers coated with immune cell membrane, it is essential to consider the state and cellular source of immune cells, since they undergo different modifications throughout the pro- and post-inflammatory phases (e.g., pro- and post-inflammatory macrophages M1 and M2).

While obtaining the desired membranes is still an attractive approach, it is becoming increasingly favorable to modify the cell surface using proteins, peptides, or small molecules before harvesting the membranes [45]. In this scenario, cell membrane receptors are becoming less sensitive, and this is unknown at this time. In the case of highly biotinylated membranes of erythrocytes, they are more likely to be taken up by macrophages because of the presence of C3b proteins on them. It is suggested that biotinylation may also disable complement regulators or self-markers on the cell surface [46]. As CMCNs appear to have no significant effect on cellular behaviours, they do not entirely reflect what the cells naturally do. Stephan et al. performed a detailed investigation of nanocarriers-tethered T cells to monitor synapse formation, transmigration, antigen, and cell division. They found that the ability of the cell to perform physiological functions was not affected by the conjugation of nanocarriers to the cell membrane [40]. The degree of immune response variability is proportional to the variety of different sources employed in cell membrane engineering and to the technology used to design the membranes. To successfully apply biomimetic-based drug delivery applications to the clinic, it is essential to have extensive CMCNPs characterization and qualification.

### Cell membrane extraction

In order to successfully isolate the cell membrane, cell membrane extraction protocols must ensure that there is minimal or no cytosol, mitochondrial, or nuclear contamination. Making use of a pure cell membrane improves surface coating efficiency and uniformity, allowing for maximum functional and structural replication on the nanocarrier surface. To preserve membrane proteins from degeneration, the extraction medium is supplemented with phosphatase/protease inhibitor cocktails that are stored at ice-cold temperatures. Prior to extraction, cells are thoroughly cleaned with saline buffer to remove any remaining remnants of the cell culture medium.

Some cells lack nuclei (e.g., RBCs and platelets), making membrane extraction easy. During membrane extraction, cells are separated first from their tissues using the most suitable techniques. For RBCs, a hypotonic treatment certainly disintegrates the cells and frees the cell membrane to collect through centrifugation in the form of a pink RBC pallet [47]. Again and again, centrifugation purifies the pallet from haemoglobin impurities. For platelets, it is recommended to do multiple freeze–thaw sequences to rapture their membrane by breaking ice crystals to release the cytosol [48]. The free cell membrane is then collected through centrifugation. Sometimes, the collected platelet membranes are treated with a discontinuous sucrose gradient to purify the platelet membrane from any high-density granules, proteins, and intact platelets.

Extraction of the membrane from nucleus-containing cells is slightly more difficult than from nucleus-free cells. Nucleus-containing cells include β-cells, fibroblasts, cancer stem cells, and immune cells (e.g., T cells, NK cells, neutrophils, monocytes/macrophages). These cells can be isolated from established cell lines like MCF-7, 4T1, J447, NK-92, etc., or from blood or tissues (stem cells, cancer cells, T cells, neutrophils, NK cells, etc.). By combining hypotonic treatment with physical disruption procedures, it produces an extract that contains high-density granules, intact cells, and free cell membranes. Finally, the cell membrane is isolated from the mixture through the use of discontinuous sucrose gradient ultrafiltration or differential centrifugation [49, 50].

Membrane functional components such as cholesterol (making structural components), carbohydrates (cellular recognition components), and transmembrane proteins (adhesion and signaling components) can be lost during membrane isolation. Cholesterol helps keep the cell membrane rigid. This loss may reduce the membrane’s mechanical stability. Moreover, proteins also act as membrane skeleton stabilizers by selectively attaching to the junction complex as well as other membrane proteins such as tropomyosin [51]. Therefore, hypotonic buffers containing divalent ions (such as MgCl_2_) or even adding cholesterol can be effective in reducing protein loss while maintaining membrane stability [52]. Moreover, the right pH, soft rapturing procedures, proper ice-cold conditions, and mild lysis buffer must be adopted for membrane extraction to avoid denaturation of transmembrane proteins/receptors. Once the cell membrane has been isolated, it is freeze dried and kept at − 80 °C to ensure that membrane proteins retain their long-term consistency and features.

### Choice of template

A template is a structural component of the CMCNs which can be used for diagnosis and drug delivery due to its various desirable features. Templates can be classified as organic and inorganic, where liposomes, gelatin, and PLGA are organic templates, while inorganic templates include iron oxide (Fe_3_O_4_), gold, mesoporous silica, upconversion nanoparticles (UCNPs), PLNPs, and MOFs. Organic templates are simple to use and provide benefits, including biocompatibility, biodegradability, and nontoxicity [53]. Inorganic templates, on the other hand, have electrical, optical, and magnetic properties that influence their selection in a CMCN [54].

For clinical translation, template biodegradability and biocompatibility are critical which are influenced by the degradation and byproducts formation and their subsequent interactions with human body. 231,231 Renal clearance helps avoid the templates adverse effects [55]. FDA-approved templates are regarded the safest in terms of toxicities. Because most organic templates are safer than inorganic ones, they have been practiced in clinical trials [56]. In 2011, a PLGA nanoparticle was used as a template to build these imitating systems [15]. As a synthetic polymer, PLGA can be fabricated into nano and microparticles and have been commonly used for RBC, platelets, cancer cells, neutrophils, dendritic cells, macrophages, cardiac stem cells, and various other templates [47, 49, 57–60]. Gelatin, a natural polypeptide widely used in medicines, food, and cosmetics, has also been utilized for assembly of CMCNs. Patient-derived tumour cells, T-cell, stem cell, and RBC are employed to coat gelatin templates for CMCNs [61–64]. Liposomes have also been used as core for cancer cells, RBCs, and macrophages membranes [22, 65, 66]. Perfluorocarbons (PFCs) are also among the regulated templates where PFCs (Fuosol-DA) was approved in 1989 but was withdrawn from market shortly due to storage issues [67]. However, PFCs are biocompatible, biodegradable, and have high oxygen-carrying capacity with ~ 20 times greater than water thus can be used for oxygen delivery to smallest capillaries and hypoxic tumour locations [68].

The toxicity of inorganic templates depends on the type of utilized metal and its breakdown in vivo. Silica is the safest (FDA-approved) inorganic template and is biodegradable and biocompatible [69]. It has been a research focus for templates due to certain properties including high surface area, porosity, and drugs or photosensitizers loading capability [70]. CMCNs have been reported using spherical silica nanoparticles on RBC, cancer cells, and macrophage membranes [71, 72]. Mesoporous silica nanoparticles can be tuned and chemically modified into various shapes and sizes for desired applications, e.g., prolonged antibacterial property and regulating endogenous reactive oxygen species for oxidative treatment [73]. When combined with CMC mimics, these tunable features could offer therapeutic benefits. The surface charge of silica templates can be changed with 3- aminopropyl triethoxysilane for CTC detection [74, 75]. Iron ions are harmless biodegradation products of Fe_3_O_4_ nanoparticles. MSCs and HeLa cells were employed as membranes in several CMC mimics employing Fe_3_O_4_ templates [76–78]. MOFs are 3D structures generated by the complexation of organic ligands and metal ions [79]. Their toxicity is associated with the type of organic linkers and metals employed. For example, zinc-based MOFs [zeolitic imidazolate (ZIF-8)] degrade to release Zn^2+^ ions, an endogenous element with a less detrimental effect on the human body [80]. Post-degradation of TPP-based Gd/Zn MOFs releases gadolinium (Gd^3+^) and zinc (Zn^2+^) ions, where Gd^3+^ is harmful to the kidneys and can pass the blood–brain barrier to accumulate in the brain [81]. Due to their structural arrangement, MOFs have excellent porosity, surface area, and photosensitizer loading capability. Gold microparticles are another inorganic template, but they are not biodegradable and may be harmful thus, nano or ultra-small templates of gold for fast renal clearance is ideal [82]. Gold particles can be shaped into nanoparticles, nanoshells, nanorods, and nanocages, which are all used to fabricate CMCNs.

## Procedures for engineering CMCNs

### Preparation of CMCNs

**Fig. 2:**
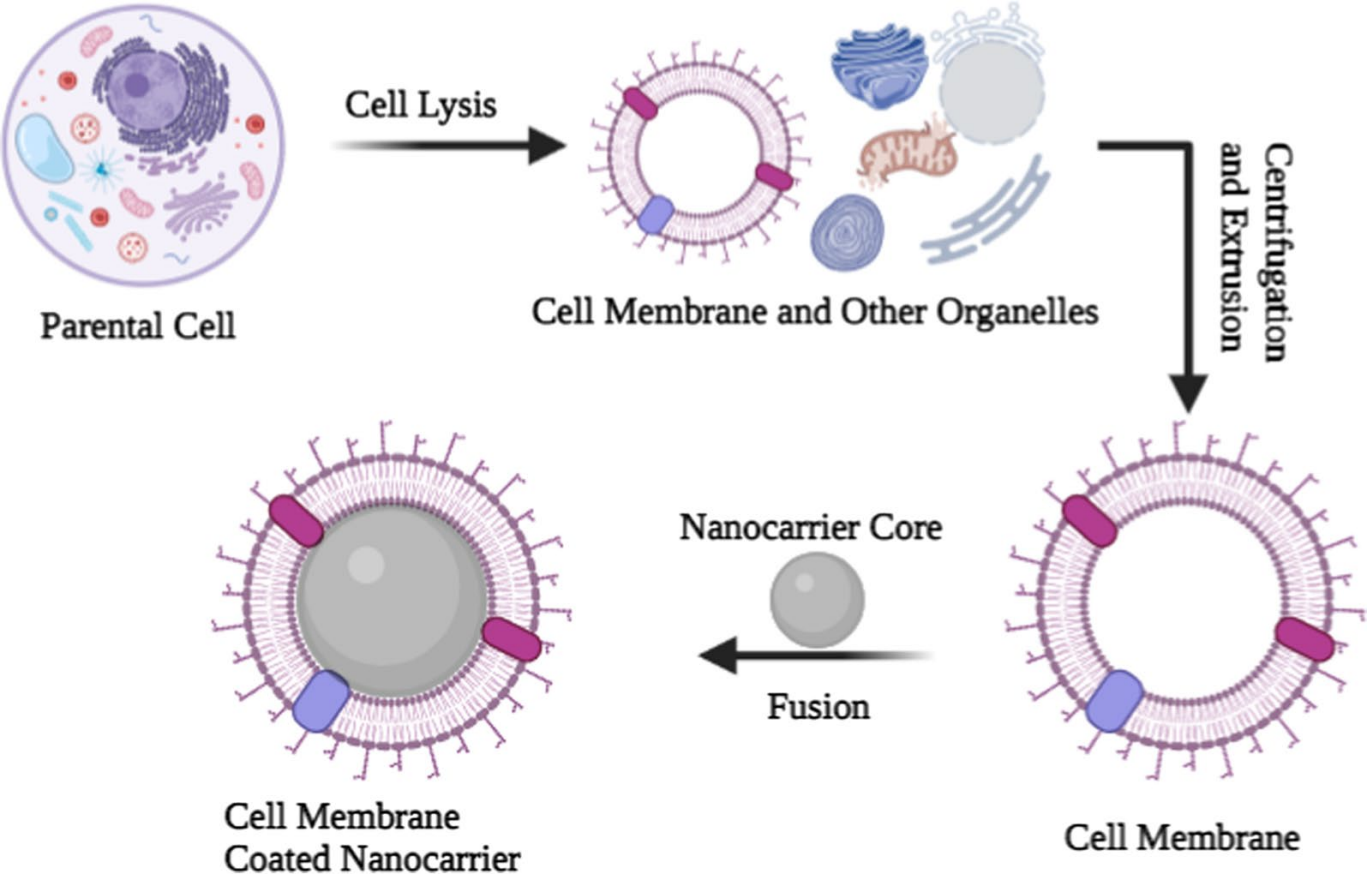
The preparation of cell membrane-coated nanocarriers is a multistep process. Cell membranes are typically synthesized in three steps: cell lysis, membrane separation, and extrusion to obtain homogenous cell membrane vesicles

The preparation of CMCNs can be processed through four major steps. The first step is to separate the membranes from the parent cells by using a hypotonic buffer to lysate them. Second, the purification of the mixture to separate cellular components and cell membranes by centrifugation [83]. The centrifugation process will be different depending on the cell type. For example, irregular sucrose gradient centrifugation is needed to prepare eukaryote cell membranes because this treatment separates the membrane from nuclei and other cell components. Whereas nuclei-free membranes like RBCs do not require this treatment. Third, preparation of the inner core. Liposomes, gelatin, PLGA, poly (-caprolactone), iron oxide nanoparticles, gold nanoparticles, mesoporous silica nano-capsules, silicon nanoparticles, and other synthetic materials make up the inner cores. The inner core selection for CMCNs is based on the types of cargo to be transported (Fig. 2).

To prepare CMCNs, the inner core nanoparticles and the cell membranes are fused together. The fusion process must be carried out in such a way that it should not result in protein denaturation or drug leakage. The two most frequently used procedures for the fusion of the inner core into cell membranes are ultrasonic treatment and membrane extrusion [84, 85]. Sonication has been employed to fuse the PLGA core into the platelet membrane, which exhibits various “self-recognized” proteins [86]. The duration, power, and frequency of the sonication should be adjusted to minimize drug leakage and protein denaturation and enhance fusion efficiency. In membrane extrusion, membranes are extruded using a technique known as sequential extrusion. In this technique, samples (a mixture of core nanoparticles and cell membrane) are extruded through different-sized pores. It is crucial to control the nanoparticle-to-cell membrane ratio in order to ensure complete surface coverage for both of these techniques [87]. A new microfluidic electroporation-based procedure has recently been developed to apply a full membrane coating on the inner core, which means that different factors, such as flow velocity, duration, and voltage, must be tailored to meet the desired results [88].

### Modification of cell membrane

The structure, functions, and components of cell membranes have become more understandable as cell biology progresses [89]. The composition of the cell membrane is mainly composed of a lipid bilayer, while protein and carbohydrate molecules are lodged in the hydrophobic part of the lipid layer [90]. One of the major functions of the cell membrane is to protect the intracellular organelles, which transport nutrients, process waste, and regulate metabolism. Moreover, cell-to-cell contacting signaling is also regulated by the cell membrane. Therefore, the cell membrane can be modified for desirable functions. The modification of the cell membrane may be processed either before disrupting the parent cells (i.e., pre-modification) or additional components are subsequently introduced into membranes after isolation (i.e., post-modification).

In pre-modification, the properties of the parent cell membrane are modified at metabolic or genetic levels. Parent cells are treated with certain chemical or physical stresses to induce the expression of specific lipid or protein components, or to modify the structure of membrane hydrocarbon chains [91]. Metabolic glycosylation is mainly used to control expression levels of native glycans, but it can also be used to introduce artificial monosaccharides into glycol-conjugates [92]. RBCs are one of the most frequently used sources for generating vesicles derived from cell membranes. However, it is impossible to modify mature RBCs genetically due to the absence of nuclei in mature RBCs. To overcome this problem, Lv et al. used the CRISPR gene-editing strategy to engineer an RBC membrane expressing the tripeptide Asn-Gly-Arg (NGR) [93]. Transgenic mice were generated in this study by inserting NRG peptide coding in the pre-embryo stage. A genetic analysis of newborn mice was used to validate the NGR expression. RBCs were isolated from these mice and used to generate RBC membrane vesicles for targeted delivery of an oncolytic virus to tumors. As exogenous physical or chemical coupling onto vesicles may alter protein function, so genetic engineering allows the biosynthesis of certain proteins using the parental protein machinery.

**Fig. 3:**
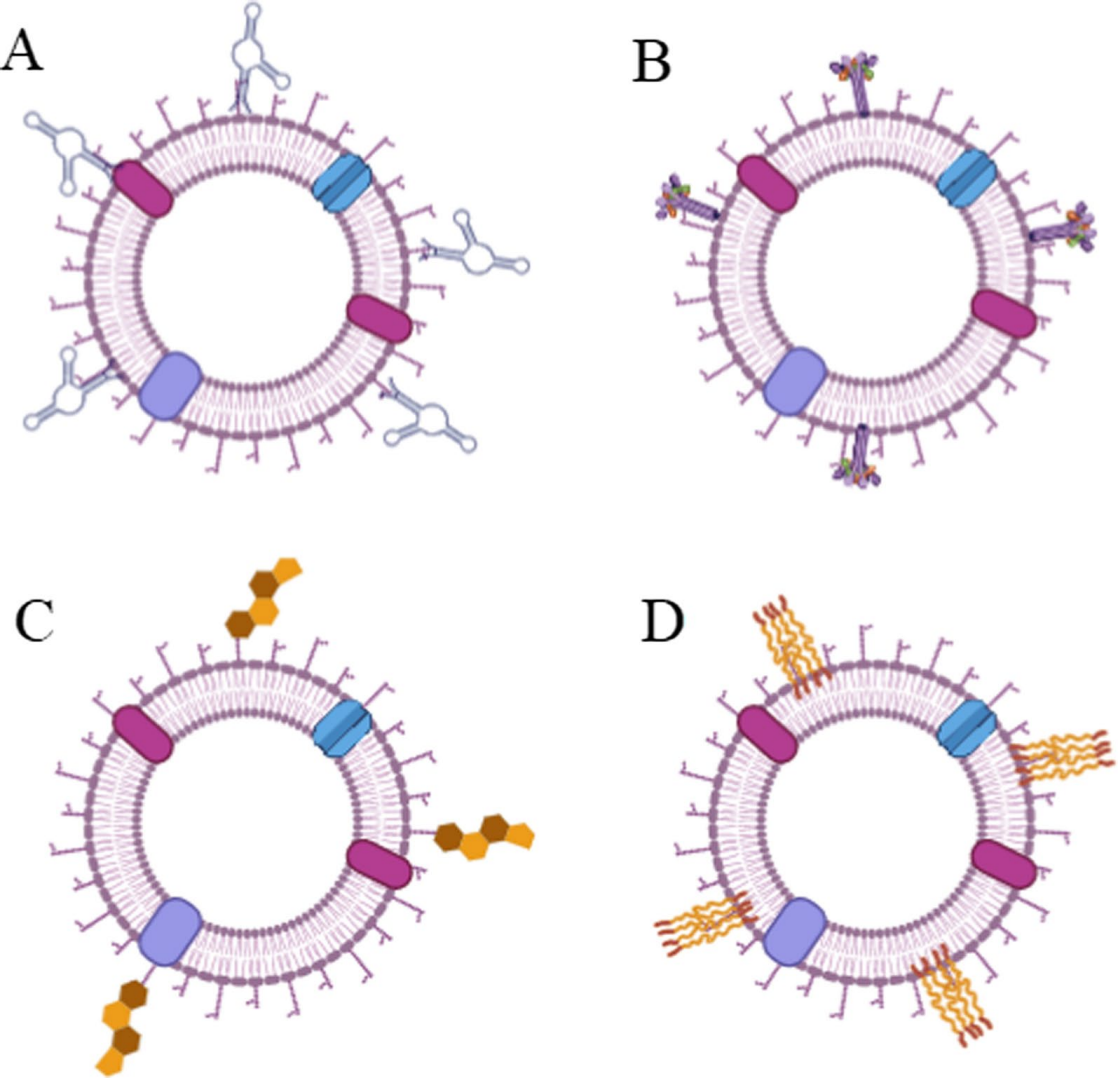
Post-modification of cell membrane. Cell membranes can be modified with different molecules or biomarkers to modulate their biological behaviors. An illustration of aptamers (**A**), protein (**B**), cholesterol (**C**), and synthetic polymer (**D**) conjugated cell membrane

Pre-modification approach results in a more homogenous and secure source of membrane, but the types of ligand and component possibilities are inadequate as compared to the post-modification approach. Several post-modification methods have been developed due to the availability of divers and convenience modified materials. The materials used for modification range from natural lipids [94], nucleic acids [95], and proteins [96], to synthetic components [97]. Cholesterol is one lipid that is used to modify vesicles derived from cell membranes for CMCNs. It plays an important role in the formation of the cell membrane’s lipid bilayer structure. Changes in cholesterol ratios can affect the rigidity and fluidity of membranes [98]. The addition of cholesterol increases the stability of vesicles derived from cell membranes in terms of their resistance to environmental pH changes [94]. In the case of RBCs, adding cholesterol to RBCs and slightly heating them for 10 min increased the rigidity of their vesicles, significantly improving the efficacy of drug loading. Proteins can be conjugated to the cell membrane through insertion or conjugation. For instance, a bifunctional linker functionalized with *N*-hydroxysuccinimide at one end and maleimide at another terminal was used to conjugate hyaluronidase to the RBC membrane [96]. Another study used an amphiphilic lipid to anchor protein to the surface of a membrane vesicle [97]. In this approach, streptavidin was first attached to 1,2-distearoyl-sn-glycero-3-phosphoethanolamine-*N*-[maleimide (polyethylene glycol)-2000] and then the lipid tail was inserted into the cell membrane. Inserting a protein-conjugated lipid into the cell membrane enables protein affixing without disturbing membrane surface proteins, thereby increasing the likelihood of membrane proteins retaining their intact structure. However, chemical conjugation of lipid moieties with a protein may alter the configuration of associated proteins. Strategies for conjugating proteins with lipid moieties at specific sites must be carefully designed to minimize possible configurational changes. Another substance used to modify the vesicles derived from the cell membrane is nucleic acids. Aptamers are short single-strand oligonucleotides that may precisely attach to a target substrate. Peng et al. used the 26-mer G-quadruplex oligonucleotide AS1411, which binds to nucleolin, to modify the membrane of cancer cells [95]. The AS1411 aptamer enabled tumor-targeting of membrane vesicles because of the overexpression of nucleolin in tumor tissue (Fig. 3).

It is well known that synthetic polymers, particularly PEG, have been used to modify cell membrane for the preparation of CMCNs [99, 100]. By protecting CMCNs from phagocytosis, PEG conjugation increases their colloidal stability and thus their circulation half-life in vivo. PEGylation can be accomplished merely through the incubating PEG lipid derivatives and cell membrane at 37 °C. PEG lipid tails easily insert into membrane layers in these conditions. One issue with current PEGylation methods for membrane vesicles is the lack of precise quantification. The outcome of PEGylation may be dependent on the compactness of PEG on the vesicles. As a next step, researchers should establish standard procedures for PEGylation of the cell membrane.

### Cell membrane hybridization

It is possible to create a hybrid cell membrane by fusing two parent cell membranes. These cell membranes have both parental cell membrane properties. Hybrid cell membranes can synergistically carry out complex behaviors. Several studies have used hybrid cell membranes to coat synthetic nanocarriers [101, 102]. For example, RBC membrane-coated nanocarriers can avoid reticuloendothelial clearance because they express CD47 (immunoregulatory marker) [103]. Similarly, P-selectin is a ligand of the CD44 receptor found in platelet membranes, allowing it to be targeted at cancer cells [104]. These membranes can be used to coat nanocarriers to improve drug delivery efficiency. It was discovered that PLGA nanocarriers coated with RBC-platelet membrane have a longer blood circulation time and better binding to MDA-MB231 breast cancer cells than plain PLGA nanoparticles [103]. Another study used homotypic targeting by fusing cancer cell membranes with RBC membranes [105]. The hybridized MCF7-RBC membrane-coated nanocarriers were found to be highly effective in terms of photothermal effect and accumulation at tumor site in MCF7 tumor-bearing mice. This study established that the protein proportion of dual membranes was a significant predictor of homotypic impact and blood retention. To achieve the best performance, the ideal proportion of the two membranes must be found empirically. Not only may hybrid membranes be formed by fusing two cell membranes together, but they can also be formed by fusing a cell membrane and a liposome together. Pitchaimani et al. reported a nanocarrier coated with a hybridized membrane of the natural killer cell membranes and liposomes [106]. The hybridization of liposome membranes in this approach enables the incorporation of several lipid components of liposomes into the cell membrane.

## Pure cell membrane used in nanocarrier coating

### Red blood cells

**Fig. 4:**
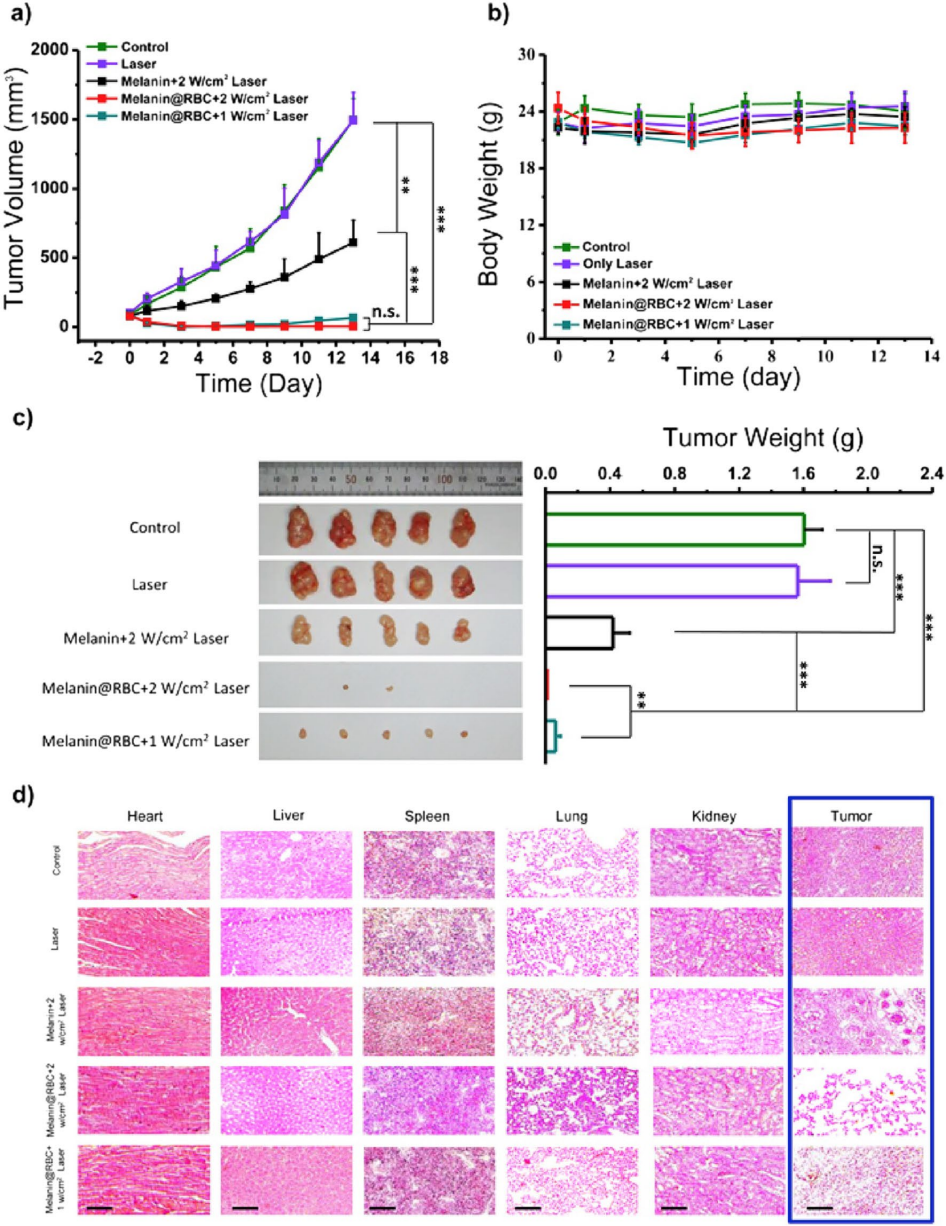
**a** Curves of tumor volume in A549 tumor-bearing mice treated with various agents. **b** Curves of body weight of mice in each group. **c** Images of tumors dissected on the 13th day following photothermal treatment, as well as a comparison of each group’s tumor weight. **d** Hematoxylin and eosin staining images of major organs and tumor tissues dissected on the 13th day following photothermal treatment. Reproduced with permission from [116]

RBCs have attracted considerable interest as a biomaterial for nanocarriers coating [83]. In humans, RBCs have a short lifespan of up to 120 days. This short-lived property of RBCs makes them an excellent source of membrane for coating nanocarriers. RBCs have a significant role in the removal of pathogens from the body via oxycytosis during the transportation of oxygen [107]. RBCs also express the ‘don’t eat me’ marker CD47, which binds to the macrophage-expressed signal-regulatory protein α preventing it from being taken up [108]. Therefore, the use of an RBC membrane to coat the nanocarrier improves the detoxification process, the removal of pathogens, and long-term circulation. Because of these properties, the RBC membrane can be used for coating a variety of nanocarriers to deliver drugs targeting breast cancer and colon cancer [108–111]. However, RBC membrane can also be functionalized with iRGD peptide and folate receptor to target breast cancer [112, 113]. For targeting the brain, targeting ligands such as T7, cRGD peptide, ^D^CDX peptide, and NGR peptide are incorporated into the RBC membrane [97, 114, 115]. Coating nanocarriers taking in anticancer drugs, photodynamic or photothermal agents with RBC membranes can be used to address the problem of short blood retention time. Recently, a study reported melanin nanocarriers coated with RBC membrane for effective photothermal cancer therapy [116]. They observed that melanin nanocarriers coated with RBC membrane had higher photo thermal efficacy in vivo than bare melanin nanocarriers due to improved blood retention and tumor site accumulation. RBC membranes have also been coated on iron oxide nanomaterials capable of photothermal conversion [111]. The iron oxide clusters coated with RBC membrane retain their photothermal properties while being less absorbed by macrophages. After intravenous injection, iron oxide clusters coated with RBC membrane showed less liver distribution and more tumor accumulation in mice. Mesoporous nanocarriers encapsulating doxorubicin have also been coated with RBC membranes for photochemotherapy of cancer [117]. Plain mesoporous nanocarriers have a short half-life and nonspecific macrophage uptake. To fight against cancer, the RBC membrane coating decreases non-specific uptake and increases blood circulation time while combining phototherapeutic and chemotherapeutic effects (Fig. 4).

Modification of RBC membranes with specific ligands can improve delivery to target tissues. For example, when RGDyK peptide was inserted into the RBC membrane used for coating of drug nanocrystals, they had a better distribution to tumors and antitumor efficacy than nanocrystals coated with unmodified RBC membrane or plain nanocrystals [97]. The CDX peptide derived from the neurotoxin was also used for the modification of RBC membranes to target brain tissues [115]. The CDX peptide was anchored to RBC membranes by streptavidin–biotin. In a glioma mouse model, CDX peptide added to RBC membranes increased brain delivery. Zhou et al. chemically rooted hyaluronidase onto the surface of RBC membranes via bifunctional linker succinimidyl-[(*N*-maleimidopropionamido)-polyethyleneglycol] ester to improve tissue penetration [96]. The modification of RBC membranes did not affect their pharmacokinetics and hyaluronidase also showed its activity as usual.

### White blood cells

**Fig. 5:**
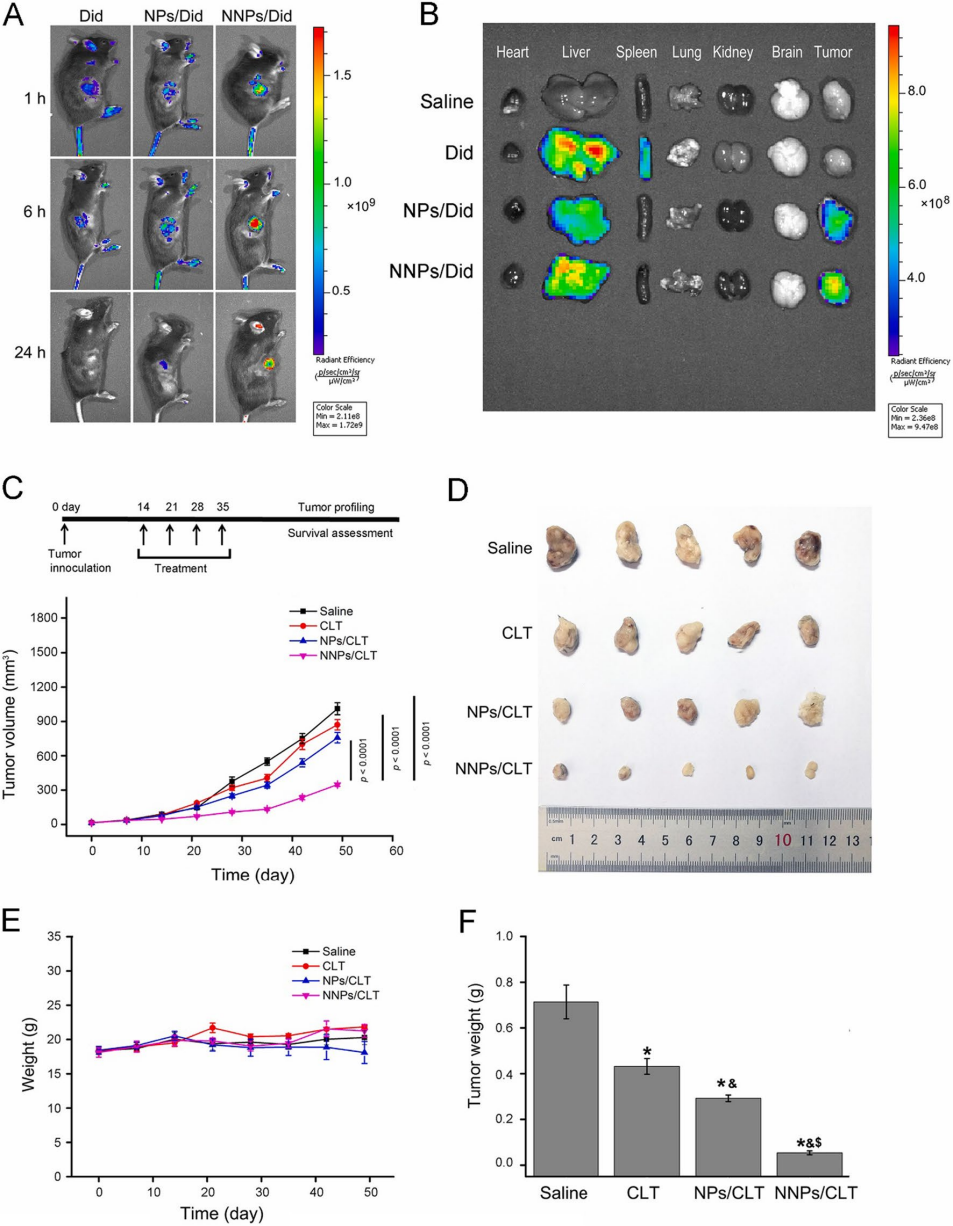
Antitumor efficacy of neutrophil membrane-coated nanocarriers in mice. **A** Images of mice after i.v. injections of Dil stain, Dil stain loaded PEG-PLGA nanocarriers, neutrophil membrane-coated Dil stain loaded PEG-PLGA nanocarriers. **B** Images of major organs and tumors after i.v. injections at 24 h, **C** average tumor volumes following various treatments over time, (**D**) morphology of tumors after 35 days, (**E**) variations in body weight, and (**F**) variations in tumor weight each treatment group over time [123]

White blood cells (WBCs) are colorless, nucleated spherical blood cells that influence disease progression. Nanocarriers surface engineered with a WBC membrane have been widely used as anticancer drug carriers in recent years due to their immune escape and active targeting abilities [118]. The most used WBCs for surface engineering of nanocarriers are neutrophils and macrophages. Neutrophils are the first immune cells to respond to tumours or infection and are closely linked to tumor progression, making them ideal carriers of antitumor drugs. They are activated by chemokines or cytokines like interferon-gamma, interleukin 8, granulocyte–macrophage colony-stimulating factor, and tumour necrosis factor α which direct them to the inflammation or infection site [119]. It has been shown that conformational variations in integrins such as L-selectin, P-selectin, macrophage-1 antigen, LFA-1, and VLA-4 also support neutrophil mobilization via extravasation from blood vessels [120]. Therefore, the neutrophil membrane can be used for surface engineering of nanocarriers to target breast cancer, circulating tumour cells, lung cancer, and premetastatic niches [25, 50, 121]. Zhao et al. reported a biomimetic nanocarrier (PTX-CL/NEs) prepared by coating PTX-loaded liposomes with neutrophil membranes [122]. PTX-CL/NEs successfully target tumor sites, release drugs, and inhibit tumor growth and recurrence. Cao et al. surface engineered Celastrol-loaded PEG-PLGA nanocarriers with neutrophil membranes [123]. Coating with neutrophil membranes allowed nanocarriers to pick up by chemokines, pass through the blood-pancreas barrier, and reach the tumor site. Although neutrophil membranes are rich in targeting content and fast to pick up by chemokines, they are preferred to use in acute treatment situations [124]. In the case of allogeneic blood as a source of WBC membranes, infectious disease screening and blood type compatibility are required because the WBCs are extremely diverse [125] (Fig. 5).

Macrophages are classified as M1 or M2 depending on their activation state. M2 macrophages reduce inflammation, suppress the immune system, and promote tumour growth, whereas M1 macrophages cause inflammation, stimulate the immune system, and extinguish tumour tissue [126]. M1 macrophages’ antitumor effect is derived from surface markers like CD86, CD80, and MHC-II. Therefore, macrophage membrane-coated nanocarriers have been widely employed for developing antitumour nanocarriers targeting breast cancer and lung metastasis of breast cancer [22, 35, 127]. The microenvironment of cancer affects macrophages, so their antitumor effect is often enhanced by administrating macrophages with other therapies. Hu et al. synthesized biomimetic nanocarriers [(C/I)BP@B-A(D)&M1m] that were encapsulated in the M1 macrophage membrane [128]. Numerous molecules involved in over expression of major histocompatibility complex (MHC) and costimulatory signal transduction on the cell membrane enabled (C/I)BP@B-A(D)&M1m to target cancer tissues effectively. When combined with laser irradiation, (C/I)BP@B-A(D)&M1m efficiently released drugs at the site of application. Liu et al. synthesized a mixed micelle containing bilirubin (ROX-responsive) and chlorin e6 (photosensitizer), loaded with paclitaxel dimer, and wrapped into a macrophage membrane. By co-delivering paclitaxel dimer and Ce6, these nanocarriers effectively combine photodynamic and chemotherapy therapy. Macrophage membranes can shield drugs from being taken up by macrophages, which increases the likelihood of nanocarriers being absorbed and retained by tumor cells.

### Platelet

Platelets are nucleate cells of blood produced by megakaryocyte fragmentation and are involved in tumor metastasis, thrombosis, and blood coagulation [129]. Platelet membranes have the ability to escape phagocytosis in systemic circulation. Like RBCs, the platelet membrane has CD47 receptors. CD47 receptors interact with regulatory proteins that inhibit macrophage receptors and can affect the pharmacokinetics of encapsulated drugs. Platelet glycoproteins may also interact with collagen-rich plaque [130], assisting in the targeting of atherosclerotic sites by platelet membrane-coated nanocarriers. So, platelet membrane coating enables nanocarriers to escape from macrophages and selectively bind injured vessels and tumour tissues. Because of these properties, platelet membrane coated nanocarriers can be used to target breast cancer lung metastasis and circulating tumour cells [57, 75]. When using nanocarriers coated with platelet membranes, it is suggested to focus on CD47 receptor integrity. A functional change in the CD47 receptor may affect biodistribution and pharmacokinetics of nanocarriers. Nanocarriers coated with platelet membranes should not be used in patients with autoimmune diseases. Platelet autoantibodies may form immune complexes with nanocarriers [131].

In recent years, the number of platelet membrane coated drug delivery systems has increased rapidly due to their easy extraction, purification, and accumulation at cancer sites [132]. Rong et al. reported a nanocarrier of platelet membrane coated black phosphorus quantum dots carrying hederagenin (PLT@BPQDsHED) [133]. PLT@BPQDs-HED had a stronger fluorescence signal at the cancer site and a higher retention rate than the control group after 48 h. A higher efficiency of drug delivery is achieved by PLT@BPQDs-HED because selectin on the platelet membrane specifically attaches to the CD44 receptor overexpressed in cancer tissue. Platelets are much more related to cancer cells, and the nanocarriers that are wrapped into platelet membranes avoid clearance by the immune system and specifically target cancer tissue via the proteins on the membrane surface. Platelet membrane-coated drug delivery systems have the potential to be used in combination with immunotherapy and phototherapy. Wu et al. wrapped nanocarriers comprising the anticancer drug and polypyrrole into platelet membranes [134]. Platelet membrane enables the drug delivery system to escape from immune systems and target the cancer tissue, laser irradiation triggers polypyrrole to cause hyperthermia and ablate the cancer cells, and anticancer drugs are also discharged from the nanocarriers to destroy the cancer tissue.

### Cancer cell

Cancer is described as abnormal cell growth that could lead to metastasis. Cancerous cells’ membrane display a variety of tumour-specific adhesion and antigen moieties. There are a wide range of molecules involved in cell–cell and cell–matrix adhesion, such as mucoprotein-1, epithelial adhesion moieties, lymphocyte-homing receptors, galectin-3, integrins, and cadherins [36, 135, 136]. Cancer cells possess properties that collectively serve a self-protective function, such as homotypic cell adhesion and immune system evasion [137]. Since these cells have unique characteristics, their membranes have gained popularity as coating stuff for nanocarriers. The dispersed membrane of cancer cells on nanocarriers allows various characteristics of cancer cells to be introduced to the nanocarriers for targeting homotypic tumours and developing personalized cancer therapy [66, 138]. Metal oxide nanoparticles, gelatin particles, mesoporous silica, and PLGA nanocarriers have all been wrapped into cancer cell membranes and used to deliver anticancer drugs [24, 72, 139, 140].

Membranes of cancer cells have the feature of homologous targeting that can be used for targeting homologous toumors [24]. In this respect, iron oxide nanocarriers were coated with HeLa cell and UM-SCC-7 membranes. When these coated nanocarriers were allowed to incubate with HeLa, HepG2, UM-SCC-7, and COS7 cells. The coated nanocarriers showed a high affinity towards HeLa cells and UM-SCC-7 cells. They could also self-target a homologous tumor and effectively restrain tumor growth in vivo. Some studies also reported the blood–brain barrier crossing ability of cancer cell membranes [141, 142]. For example, nanoparticles of polycaprolactone/F68 were coated with secondary brain cancer cell membranes and then loaded with indocyanine green, a photothermal and imaging agent [141]. Intravenous injection of these nanocarriers into mice bearing U87MG-Luc glioma cells showed high distribution in the brain. Similarly, PEG-PLGA nanocarriers coated with MDA-MB-831 cancer cell membrane were investigated for use in treating brain cancer [142]. They found that the accumulation of coated nanocarriers in the brain was higher than uncoated nanocarriers.

### T cells

T cells play an important role in adaptive immune responses [143]. T cells need antigen priming through a specific T-cell receptor (TCR) for activation. The dendritic cells (DCs) possess the MHC-antigen complex that engages with the TCR and activates T cells. Activated naive T cells become regulatory or effector T cells, depending on the DC-T cell immune synapse context. Effector T cells scavenge and kill cancerous or virus-infected cells in the bloodstream. Moreover, T cells can also mature into memory T cells, which offer long-lasting protection against foreign bodies that activated them. Therefore, T cell membranes coated nanocarriers can be used to target gastric cancer, liver cancer, and tumour tissues [85, 92, 144].

T cell membranes were used to wrap PLGA nanocarriers loaded with dacarbazine [145]. In this study, T cell membranes were extracted from the EL4 cell line and incubated with PLGA nanocarriers loaded with dacarbazine. T cell membrane-coated nanocarriers were able to bypass tumor immune suppression and neutralize TGF-b1 and PD-ligand 1 expression in the tumor environment. Furthermore, T cell membrane-coated nanocarriers improved dacarbazine delivery and enhanced apoptosis of tumor cells. Ma et al. reported the development of a nanocarrier composed of mesoporous silica holding IR780 nanoparticles wrapped into the membranes of chimeric antigen receptor T cells (CAR-T) to exclusively target hepatocellular carcinoma cells (HCCs) expressing GPC3 [85]. They engineered the CAR-T cell nanocarrier in such a way that it could detect GPC3-expressing HCCs. The results demonstrated that NP-coated CAR-T cell membranes were more effective at targeting HCC cells in vivo and in vitro than IR780-loaded mesoporous silica. CAR-T cell therapy is a newer blood cancer treatment. Ex vivo CAR-T cells are produced by genetically modifying TCR to recognize an antigen without antigen presentation. Ex vivo-amplifier CAR-T cells are then reinfused into hematological cancer patients. The FDA has approved CAR-T cell targeting of the CD19 antigen for the treatment of relapsed/refractory diffuse large B-cell lymphoma or acute lymphoblastic leukemia.

### Dendritic cells

Dendritic cells (DCs) are immune cells that gather around cancer cells due to immune signals (such as pathogen-associated molecular patterns and proinflammatory cytokines). They transfer tumor-associated antigens to lymph nodes to establish communication with naive T cells for differentiation into attack cancer cells and mature T cells [146]. For this reason, designing tumor immunotherapy around DCs characteristics is a promising approach. However, issues like complex preparation methods, short efficacy periods, and high cost still need to be addressed [147]. DC membranes contain components like DC-originating molecules and can target and stimulate the immune systems of their source cells [148]. It has been shown that CD40/CD80/CD83/CD86 are upregulated on the DC membranes as co-stimulatory receptors [149]. The binding of these molecules to their respective receptors on T-cells activates DCs to produce cytokines such as IL-10, IL-12, and interleukin that distinguish T cells into their anti-inflammatory or pro-inflammatory subsets. Therefore, DC membranes coated nanocarriers can be used to target prostate cancer and ovarian cancer [150, 151]. Cheng et al. reported an IL-2-loaded PLGA nanocarrier warped in membranes derived from DCs [151]. The DCs derived membranes provide unique and potent stimulatory signals and sustain a strong T-cell response due to their intact surface proteins. The nano-dimensions of this carrier may be a significant contributor to the T cell response by eliminating spatial barriers throughout antigen presentation. Zhang et al. used a combination of a nanocarrier-based antigen delivery system and photochemical internalization to induce tumor-specific cytotoxic T cells in their study [152]. It was demonstrated that the combination of a hydrophobic photosensitizer (Pheophorbide A) and polyethyleneimine possessed the ability to evade endosomal degradation while also enabling near-infrared imaging. Moreover, by grafting the synthesized complex onto ovalbumin, a model antigen, light-sensitive nanocarriers were formed.

## Hybrid membrane used in nanocarrier coating

Hybrid membranes can be used to combine the properties of a variety of cell membranes and optimize their function [101]. In general, hybrid membrane coated nanocarriers (HMCNs) more specifically interact with the cancer environment, resulting in improved specific targeting, minimizing non-specific interactions with abundant proteins and cellular components, and optimizing specific biological roles [153]. Moreover, a hybrid membrane incorporates at least two distinct biological activities. One is a competence for targeting, whereas the other refers to inherent properties conferred by the membranes of a source cell. The targeting potential is primarily comprised of homologous targeted delivery to tumor sites via DC membranes and cancer cell membranes, specific tumor targeting via PLT membranes, the capability of tumor targeting enhancement via membranes of stem cells, and circulating tumor cells targeting via WBC and PLT membranes [75, 154–156]. The latter biological function types mainly include prolongation of blood circulation via PLT and RBC membranes; specific adherence to injured vessels via PLT membranes; immune evasion via PLT and WBC membranes; toxin neutralization and absorption via RBC and macrophage membranes; and activation of the immune system via bacterial outer membranes, cancer cell membranes and immune cell membranes. Due to the membrane combination, HMCNs can achieve maximum functionality in diverse biomedical fields.

The leukocyte membrane is considered a naturally occurring coating material with the biomimetic potential, capable of evading immune system capture and inflammatory targeting via inducing inflammation via specialized ligand-receptor interaction [121]. Vectors that are similar to leukocytes could continue their capabilities, such as inhibiting particle phagocytosis and opsonization, facilitating the transportation over the endothelial layer while avoiding the lysosomal pathway and thereby delaying the clearance by the liver [27]. However, a drug delivery system based on a single leukocyte membrane is incapable of achieving adequate therapeutic efficiency because of its incapability towards tumor targeting. Thus, He et al. joined a leukocyte membrane with a cancer cell membrane to increase the targeting potential of HMCNs. The leutusome was produced by fusing together the membranes of leukocytes, tumor cells, and liposomal nanocarriers simultaneously [157]. Encapsulation of paclitaxel (PTX) with leutusomes significantly reduced tumor development without causing systemic damage (in vivo), suggesting that selective taken up of leutusomes by tumor cells. After 48 h, leutusomes labeled with DIR displayed substantial fluorescence in tumor sites that were 9.3-fold larger than those in the control. The liposomal NPs accumulation from leukocytes or cancer cells was 2.7-fold and 4.4-fold more in the tumor, respectively, than in the control. Additionally, the study indicated that coating outside of the cores or incorporation into liposomal nanocarriers had no effect on the unique features of different cell membranes. These composite biomimetic nanocarriers outperform solid tumour homing and have a longer circulation time due to surface markers expressed on both cell types.

Sun et al. developed a cancer cell-RBC hybrid membrane coated gold nanocage loaded with doxorubicin to treat breast cancer via chemotherapy, photothermal therapy, and radiotherapy [158]. Homological targeting of the cancer cell membrane and reduced clearance by the RBC membrane made the HMCNs particularly effective in accumulating in tumor sites. Macrophages have been associated with the early dispersion of cancer and hence have a substantial effect on prolonging metastasis throughout the progression of cancer. Gong et al. developed a hybrid membrane composed of macrophages and cancer cells coated with doxorubicin-loaded PLGA nanocarriers for use in breast cancer treatment to specifically target lung metastases [159]. Since RAW264.7 membrane exhibits enhanced expression of high integrin α4β1, the resultant HMCNs demonstrate remarkable membrane-derived features, which include the capacity to target homologous cancer cells and improved particular metastatic targeting potential. The metastatic nodule numbers in the lung were reduced by about 88.9% after the therapy of lung metastases derived from breast cancer, which performed better than the pure CMCNs. This hybrid membrane derived platform demonstrates promise as a biomimetic nanoplatform for the metastasis treatment of breast cancer.

He and Su’s group previously described the use of HMCNs based on RBC and retinal endotheliocyte membranes for non-invasive therapy of choroidal neovascularization [160]. The RBC and retinal endotheliocyte membranes fusion provide protection to the nanocarrier against phagocytosis while also giving the potential to the HMCNs to bind with vascular endothelial growth factors, enhancing their potential to target choroidal neovascularization regions actively. particularly, the anti-VE-cadherin antibody suppressed the fluorescent signals in HMCNs-treated cells, demonstrating that the ability of self-targeting is dependent on surface binding molecules (N- and VE-cadherin) expression on the retinal endotheliocyte membrane [161]. The substantial fluorescence colocalization of angiogenic retinal endotheliocyte membranes and HMCNs in the tube formation experiment also indicated the nanocarriers’ targeting ability. Furthermore, using a quantitative examination of the mean fluorescence intensity, the group treated with HMCNs drastically decreased damage area and choroidal neovascularization leakage in contrast to the group treated with pure CMCNs in a choroidal neovascularization mouse model induced by laser. In conclusion, dual-fused membrane-based nanocarriers offer significant advantages over currently available invasive therapies.

## Challenges and future directions

Numerous advantages have been reported for CMCNs, particularly in terms of biocompatibility and targeting. Synthetic DDSs currently available are basically foreign substances with the potential for immunogenicity and toxicity. Whereas cell membranes are endogenous, they are considered biocompatible and perform a variety of biological functions like the source cell. However, certain issues must be resolved before these carriers can continue to evolve and move from the laboratory to the clinic.

The first and most important question to be addressed is about the yield of cell membranes and extracellular vesicles. Not only do existing separation technologies produce a negligible amount of cell membranes and extracellular vesicles, but they are also prohibitively expensive for large-scale production. As a result, more sophisticated large-scale manufacturing methods are required to continue expanding the application of cell membrane. In recent years, to address the yield issue, extensive work has been carried out on techniques which are used for generating artificial vesicles when the membrane is ruptured via extrusion. For example, the same number of THP-1 cells yield more than twice as many simulated exosomes as natural exosomes, and the drug encapsulating and releasing rates of the simulated exosomes are also higher [162]. The extraction and purification procedures must also be revised and optimized, as many cells must still be cultured to obtain an adequate number of membranes, and the preparation procedure must still be simplified [118]. For RBCs membrane-coated nanocarriers that lack a targeting ability, the membranes must be modified to reach the target site for therapeutic cargo release, but this will likely change the membrane’s original structure and reduce its biocompatibility. Platelet membranes are highly sensitive, so finding an appropriate loading scheme to ensure adequate drug loading and reliable delivery to the target tissue is challenging. The toxicity and stability of modified membranes must also be studied, especially as nanocarriers for cancer therapy. To achieve the desired dose and release profile, the drug loading method should be chosen carefully [163].

Moreover, a complete understanding of the mechanism of transporting cell membranes extracted from different sources in vivo is unknown and requires further research. For example, therapeutic molecules delivered by white cell membrane carriers may activate immune system components and cause inflammation [164]. When cancer cell membrane is used, it may cause cancer in the body if the parent cancer cells’ genetic material is not completely removed. Procedures for purifying and characterizing cell membranes are not consistent and differ from laboratory to laboratory, causing confusion about the physicochemical features of the cell membrane. So, it is necessary to share the scientific data and develop a standardized procedure for cell membrane quality control that is highly repeatable. Nanocarriers wrapped into cell membranes and extracellular vesicles can target cancer tissues crossing biological barriers. Some cells can be used to both extract membranes and isolate extracellular vesicles to transport drugs. While it is relatively simple to extract and prepare the cell membrane, the targeting ability may be compromised due to protein loss during membrane extraction. However, extracellular vesicles are difficult to prepare, they generally retain all membrane components, giving them excellent targeting ability [165]. As a result, the appropriate carrier must be chosen according to the experiment’s objective in order to maximize the therapeutic effect.

## Conclusions

The development of therapeutics derived from cell membrane material is a rapidly growing field of research that is particularly appealing because it involves an organic cellular networking system. Biomimetic technology has the advantage of taking advantage of the natural mechanisms of living matter, but it is also a double-edged sword. It is difficult to know which components, out of the multiple factors, confer membrane functionality, and so the ratio of each component needs to be modified as needed. To develop drug-containing membrane-coated carriers, a similarly and standardized manufacturing process will be required. Despite the difficulties associated with processing variables, manufacturing, and quality control, vesicles derived from natural cells have the advantage of being bioactive, reflecting the features of the parent cells. Although membrane-coated nanocarriers face numerous challenges, a powerful advantage of ‘mimicking nature’ overrides many disadvantages of traditional DDSs and offers a more efficient approach for cancer treatment. With the rapid advancement of nanotechnology, proteomics, bioinformatics, pharmacology, and material science, it is expected that the combination of DDSs and cells will overcome numerous obstacles, revolutionize current medical technology, and open up new avenues for targeted cancer therapy.

## Data Availability

Not applicable.
